# Comparing DNA yield from fish scales following different extraction protocols

**DOI:** 10.1038/s41598-022-06889-y

**Published:** 2022-02-18

**Authors:** Loraine Shuttleworth, Carel Jakobus Oosthuizen

**Affiliations:** Department of Zoology and Entomology, University of Pretoria, Private Bag X20, Hatfield, 0028 South Africa

**Keywords:** Genetics, Zoology

## Abstract

Studies on genetic diversity, adaptive potential and fitness of species have become a major tool in conservation biology. These studies require biological material containing a reliable source of DNA which can be extracted and analysed. Recently, non-invasive sampling has become the preferred sampling method of such biological material; particularly when studying endangered species. Elasmoid scales from teleost fish are an example of non-invasive samples from which DNA can successfully be extracted. This study compared different extraction protocols to find an optimal method for extracting DNA from teleost fish scales. This was done with the intent to use the protocol that yielded the highest quantity of DNA on dried, archived scales. The protocols tested in this study included (1) phenol/chloroform with a TNES-urea digestion buffer, (2) phenol/chloroform with an amniocyte digestion buffer and (3) Qiagen DNeasy Blood and Tissue Kit with variations in incubation times and temperatures of each protocol. While the phenol/chloroform with TNES-urea digestion buffer yielded significantly higher concentrations of DNA compared to the other protocols, all protocols followed in this study yielded sufficient quantities of DNA for further downstream applications. Therefore, while there are multiple viable options when selecting a DNA extraction protocol, each research project’s individual needs, requirements and resources need to be carefully considered in order to choose the most effective protocol.

## Introduction

The use of DNA-based studies for investigating genetic diversity, adaptive potential and fitness of threatened and endangered species has become increasingly popular in the field of conservation biology^1–3^. Data gathered from these studies can be used to inform management strategies, allowing intervention when extinction due to loss of adaptive potential is imminent^4^. Notably, adaptive potential is becoming increasingly important due to the rapidly appearing effects of human-induced climate uncertainty in addition to stochastic fluctuations of the environment^4,5^. Therefore, analysing levels of genetic diversity is considered to be integral in deciding which populations should be prioritised for protective action^2,6,7^.

In order to analyse levels of genetic diversity, biological material such as blood, muscle, feathers, or faeces, which contain a reliable source of DNA, is required^8^. There are various methods that can be used to obtain the chosen biological material with different DNA extraction techniques being more suited to certain materials than others. Recently, non-invasive sampling has become a preferred choice—particularly when studying endangered species as this limits further harm to the already stressed populations^9,10^. It should, however, be noted that there are some concerns when using non-invasive samples as a source of DNA due to the fact that they often yield low amounts and/or low quality DNA^11,12^. There are also authors who believe that the term non-invasive should be strictly reserved for samples which can be collected without having to catch or disturb the animal in any way^13^. Under this definition, fish scales would not be considered non-invasive samples as they need to be physically removed from the fish^14^. Regardless, for this study (as with many others), scale sample collection was considered to be non-invasive^10,15,16^. While the removal of scales, similar to taking fin clippings, might cause a slight acute stress response in the fish, these sampling methods are non-lethal and long-term studies suggest that they have no significant effect on fish health^17,18^.

Although challenging, scale samples have been found to yield sufficient amounts of DNA to be used with a variety of molecular techniques making them extremely valuable for studying changes in populations^19^. Dried scales are commonly stored by fishery authorities, and are often readily available to be used in research^16^. Therefore, along with an optimised extraction technique, scales as a source of DNA might become a preferential DNA source (especially when studying ornamental or endangered fish) due to the non-invasive sampling method and ease of acquisition^16^.

Teleost fish are the largest and most diverse vertebrate group and occupy nearly every ecological niche possible for an aquatic vertebrate^20,21^. Globally, teleost fish are becoming increasingly threatened at all levels of biological organisation which is proving to have significant impacts on many ecological processes^21^. Consequently, the need to study and conserve the genetic diversity of these species is essential.

**Figure 1:**
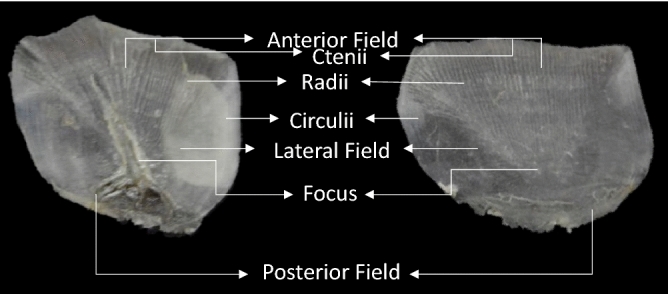
Morphology of the ctenoid scales from *Argyrosomus thorpei* (Photo by ©L. Shuttleworth, edited in MS PowerPoint Build 14527.20234).

The scales that are commonly found on teleost fish are referred to as elasmoid scales^22,23^. Morphologically, these scales can be divided into three distinct regions: anterior, lateral and posterior (Fig. 1). While the posterior region (which lies directly below the epidermis) is exposed to the environment, the anterior and lateral regions are covered by surrounding scales with the anterior region embedded within the dermis^24^.Within the anterior region are a series of radial grooves and circular ridges around the central ‘focus’^22,24^.The dermis and epidermis entirely consist of living cells^25^. Extracted DNA from dried scales would thus be sourced from dermal and epidermal cells that dried and collected within the ridges of the scale^26^.

Kabeljou (*Argyrosomus thorpei*), the species used for this study, have elasmoid scales—specifically ctenoid scales^24^.

This study aimed to optimise a protocol for DNA extraction from fish scales so that it may be applied to a limited number of dried archived scales. As previously mentioned, dried scales are commonly stored by fishing authorities. This is however often done for the purposes of short-term objectives and maintaining archived collections is therefore not a common occurrence^27^.

Successfully extracting DNA from the archived samples will enable us to greatly improve our existing knowledge of species in general. Gathering population genetic data will enable the investigation of the potential effects that a stock collapse has had on genetic diversity within a population as an example. It will also contribute to determining if the conservation measures for a specific species have been an effective tool in protecting the population and allowing for genetic diversity to be gained over time.

Two main DNA extraction methods were used in this study. These included phenol/chloroform DNA extractions, as is used in one of the most popular methodologies for extracting DNA from fish scales^28^ and DNA extractions using the commercially available Qiagen DNeasy Blood and Tissue Kit due to the ease of use when using extraction kits like this one. Variations of these extraction methods concerning incubation time and temperature were included in the methods to identify an optimized protocol for the isolation of high quantities of DNA. Lastly, the scales were divided into three regions (Fig. 6) to determine if, on average, a certain region contained a higher concentration of DNA. If this held true, the particular region could be used when extracting DNA from the archived samples as this would likely lead to the most successful extractions while the remainder of the scale could be kept and possibly used in future research.

## Results

### Scale fragmentation

Following freezing with liquid nitrogen and pulverisation, the scales did not form a powder as expected. The scales remained whole, however, noticeable markings could be seen from trying to grind the scale with the pestle. This approach did not work, therefore, scales were fragmented by cutting them into very small pieces using sharp, sterile scissors.

### DNA extraction

#### Phenol/chloroform DNA extraction

**Figure 2:**
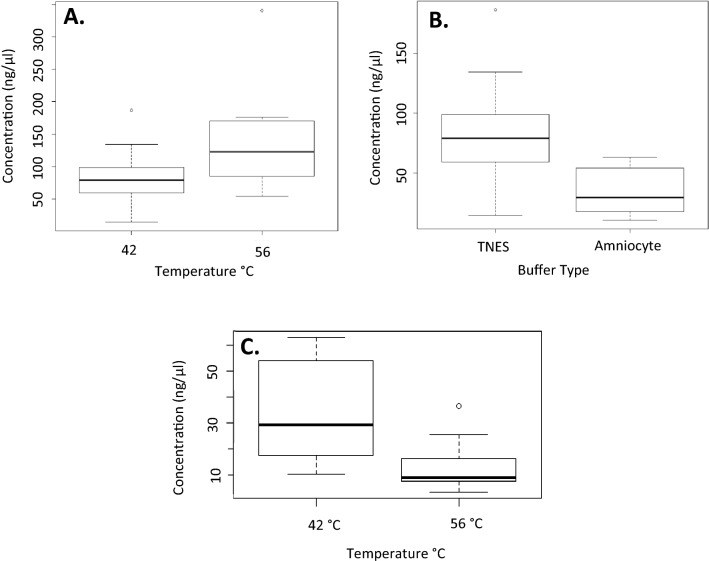
Box plots of the concentration of DNA following extraction using the phenol/chloroform protocol with TNES-urea digestion buffer and overnight incubation at different temperatures (**A**). The concentration of DNA following DNA extraction using the phenol/chloroform protocol with different digestion buffers at 42 °C overnight incubation (**B**) and the concentration of DNA following extraction using the phenol/chloroform protocol with an amniocyte digestion buffer and overnight incubation at different temperatures (**C**).

To compare phenol/chloroform Variations 1 and 2 (digestion temperature increased from 42 to 56 °C), a Wilcoxon rank-sum test (the non-parametric alternative to the two-sample t-test) was used. This showed that there was a significant difference in DNA yield when samples were incubated at different temperatures (p < 0.05). Incubation at 56 °C yielded higher concentrations on average (mean = 131.56 ng/μl) compared to incubation at 42 °C (mean = 75.57 ng/μl) (Fig. 2A) The same was done to compare Variations 1&3 (TNES-urea buffer replaced by amniocyte digestion buffer) which revealed that the TNES-urea buffer worked significantly better (mean = 75.57 ng/μl) than the amniocyte buffer (mean = 35.18 ng/μl) (p < 0.05)(Fig. 2B). The range in DNA yield for these comparison can be seen in Fig. 2.

In addition, a Wilcoxon rank-sum test was used to test how a higher incubation temperature (56 °C) would affect DNA extraction using amniocyte buffer. A Wilcoxon rank-sum test confirmed that there was a significant difference between 42 and 56 °C incubation temperatures (p < 0.05) with the 42 °C incubation temperature yielding higher concentrations of DNA (mean = 34.14 ng/ μl) than the 56 °C incubation temperature (mean = 12.40 ng/ μl ) when used with an amniocyte buffer. This result can be seen in Fig. 2C. The higher DNA concentrations for the digestion temperatures used with a TNES buffer (42 °C vs. 56 °C) can also be seen in Online Appendix A.

#### Qiagen DNeasy blood and tissue extraction kit

**Figure 3:**
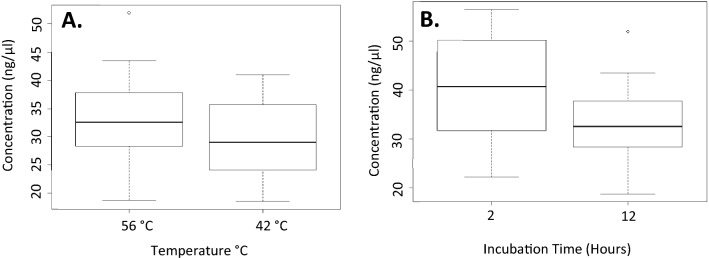
Box plots of the concentration of DNA following genomic DNA extraction using the Qiagen DNeasy Blood and Tissue Kit with different overnight incubation temperatures (**A**) and different incubation times at 42 °C (**B**).

A two-sample t-test was used to compare how both different incubation times, as well as different incubation temperatures, would affect DNA yield. This revealed that variation in neither time nor temperature significantly affected DNA yield (p > 0.05). The range in DNA yield for the kit protocol comparisons can be seen in Fig. 3.

#### phenol/chloroform vs. Qiagen DNeasy blood and tissue extraction kit

**Figure 4:**
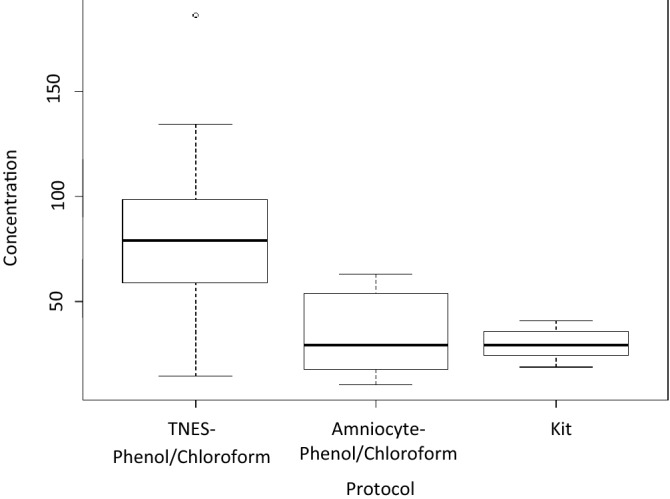
Box plots of the concentration of genomic DNA following extraction using three different extraction techniques with incubation at 42 °C for 12 h.

Post-hoc analysis (TukeyHSD, p < 0.01) of the three different genomic DNA extraction protocols with overnight incubation at a temperature of 42 °C revealed a significant difference between TNES-phenol/chloroform and amniocyte-phenol/chloroform extractions and TNES-phenol/chloroform and kit extractions (p < 0.01). No difference was observed between amniocyte-phenol/chloroform and kit extractions (p > 0.01). TNES-phenol/chloroform yielded the highest concentrations of DNA (mean = 81.73 ng/μl) followed by amniocyte-phenol/chloroform (mean = 34.14 ng/μl) and then the kit (mean = 29.66 ng/μl). The range in DNA yield between the three different protocols can be seen in Fig. 4.

#### Phenol/chloroform DNA extraction from scale regions

**Figure 5:**
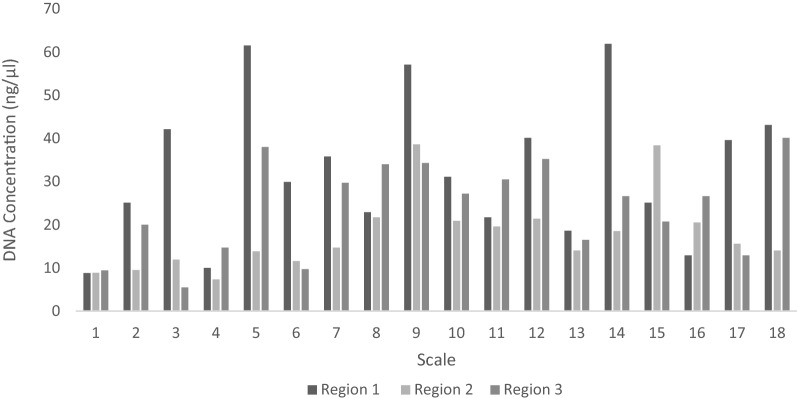
Bar graph indicating the concentration of extracted DNA from the three different scale regions. Extractions from scale 1–6, 7–12 and 13–18 were completed on three consecutive days.

From the bar graph indicating the DNA concentration across different scale regions (Fig. 5), it was observed that on average, Region 1 yielded the highest concentration of DNA. Mean values (region 1 = 32.63 ng/μl, region 2 = 17.83 ng/μl and region 3 = 23.98 ng/μl) and post-hoc analysis (TukeyHSD, p < 0.01) confirmed this observation and revealed that there was a significant difference in concentration between Regions 1 and 2. No significant differences were observed between Regions 1 and 3 or Regions 2 and 3.

### Confirmation of DNA quality and species ID

Selected samples representing each extraction technique was successfully PCR amplified in order to confirm suitability for downstream DNA application. Sequencing of a representative sample was performed. A Mitochondrial DNA COI gene fragment of 657 bases in length were obtained (GenBank accession number OM574579) and the species identity confirmed.

## Discussion

Isolation of sufficient quantity and quality DNA from samples collected non-invasively such as fish scales has become largely of interest for studying genetic diversity for conservation purposes. The identification of an optimal extraction protocol for such samples was therefore pursued in this study with the ultimate aim being that this protocol would be equally effective when used on archived scale samples in future. The preservation of these scales prompted the recognition of the value of archived samples not only for genetics studies but for other fields of research as well. Therefore, prior to DNA extraction, measures to prevent unnecessary damage to samples from which DNA could not be extracted but which might be suitable for other research types were investigated.

Scale fragmentation facilitated by liquid nitrogen proved to be unsuccessful. The structure of the scales was taken into consideration as the possible contributing factor. The collagen fibrils of teleost scales are arranged in a “plywood” structure^29,30^. These structures are characterised by discrete layers of either fibrils and/or fibre bundles arranged in a parallel orientation to one another but with a different fibril orientation between each discrete layer^31^. There are three variations of the plywood structure namely orthogonal plywood, twisted plywood and double-twisted plywood^31^. This plywood arrangement of the collagen fibrils makes fish scales much stronger and more isotropic in mechanical behaviour that indeed, many scales have been found to be unable to tear even after complete submersion in liquid nitrogen^32,33^. Although more time consuming, using scissors to cut the scales proved to be a sufficient method for fragmentation as complete cell lysis during the incubation period was achieved for each extraction protocol with its respective digestion buffer.

The overnight incubation time and 42 °C temperature of the phenol/chloroform extractions were according to the protocol set forward by Wasko et al.^28^. These authors suggested that tissues only partially digest at temperatures lower than 42 °C but that higher incubation temperatures (50 °C or more) are “inefficient”. While it was unclear exactly what is meant by the word “inefficient”, it was assumed that this could have been related to the performance of the proteinase K at higher temperatures. However, contrary to the recommendations of Wasko et al.^28^, the results of this study showed that an incubation temperature of 56 °C performed better than the suggested 42 °C when using a TNES digestion buffer as these authors did. In a study by Abubakar et al.^34^, proteinase K needed to be heated up to 98 °C for 10 min for it to be inactivated. Therefore, it seems there is no practical reason to keep the incubation temperature below 50 °C, particularly not when doing extractions from archived scales where the highest efficiency of DNA extraction will be required. It is however interesting that a 42 °C incubation temerature performed better when using an amniocyte buffer. This was contrary to the expectation that a higher incubation temperature (56 °C) would improve the DNA yield when using a “weaker” buffer.

Another suggestion by Wasko et al.^28^ was the use of a TNES buffer supplemented with additional urea as an optimal digestion buffer. The results of this study suggest that the TNES-urea buffer was a good choice to increase the yield of extracted DNA. The alternative buffer that was used, an amniocyte buffer which is often used with phenol/chloroform extractions, proved to be suboptimal. Since an amniocyte buffer is very similar to a TNES buffer this could primarily be attributed to the addition of the urea to the TNES buffer. Urea is known to break down hard tissues like scales and this is why Wasko et al.^28^ chose to add it to the extraction buffer. In addition to this characteristic, Hilz et al.^35^ showed that proteinase K activity is stimulated by urea, a factor which could further have contributed to the success of the TNES-urea buffer extractions.

While phenol/chloroform extractions were successful at isolating DNA from the scales, there are known safety hazards posed by handling phenol as well as disposal problems associated which could not be completely disregarded^36^. In addition, phenol/chloroform extractions are laborious and the multiple steps required make this method of DNA extraction highly prone to cross-contamination^37^. Commercially available extraction kits, such as the Qiagen DNeasy Blood and Tissue Kit, are much simpler to use and eliminate the need for volatile organic solvents. Therefore, they are not associated with the same extensive health risks as phenol/chloroform extractions and disposal of the reagents used in these kits is also much more convenient since special precautions do not need to be taken for waste removal. For these reasons, this study investigated whether kit extractions are viable for DNA isolation from fish scales and whether the extraction yield could compete with that of the phenol/chloroform extractions.

It was shown here that the protocol provided by the manufacturers was effective in extracting DNA from the scales. The 2 h incubation time recommended for fin clippings, is also applicable to fish scales since the DNA yield from the digestions reached a maximum within that time period. Neither adjustments to time, nor temperature had any significant difference in the DNA yield when the Qiagen DNeasy Blood and Tissue kit was used. In terms of temperature, the lower-than-recommended temperature performed similarly to the 56 °C suggested incubation temperature. Therefore, in this case, a temperature of above 50 °C might be considered “inefficient” as Wasko et al.^28^ previously stated.

After establishing that DNA could be isolated using any one of the DNA extraction protocols included in this study, it was decided to proceed with the protocol by Wasko et al.^28^ to investigate which region, if any, would likely yield the highest amount of DNA. It was found that out of the three defined regions, Region 1 yielded the highest concentration of DNA. This region consisted of the posterior field of the scale from the focus down and included the ctenii. It has been hypothesised that one of the functions of ctenii is to provide a substrate for the attachment of the epidermis and mucous layers that cover the scales^38^. Therefore, it is possible that the higher DNA yield at Region 1 could be attributed to additional epidermal cells which remained attached to the ctenii as well as those that accumulated in the ridges of the circulii. Region 3 yielded the second-highest concentration of DNA. This region included the anterior field—from the focus upwards but only the parts that included the radii. Lastly, Region 2 which included the lateral circulii covered portions on either side of the focus, yielded the lowest concentration of DNA. While both Region 2 and Region 3 are covered by adjacent scales, Region 3 is deeply embedded within the dermis^24^. It is therefore likely that the friction caused by adjacent scales removed many of the epidermal cells that could possibly have clung to Region 2 while the dermis protected Region 3 from such friction. It is then possible that some of the DNA extracted from Region 3 originated from dried dermal cells which accumulated in the radii. While the assumption that most of the extracted DNA would be sourced from dried dermal and epidermal cells could be true, Region 2 still yielded some DNA. This indicates that there was some DNA within the actual scale. Le Guellec et al.^39^ state that within the plywood collagen matrix of the scale, there are a few interspersed fibrocytes which could explain the origin of the extracted DNA.

## Conclusion

When comparing phenol/chloroform extractions using different digestion buffers to extractions using a commercially available kit with incubation at 42 °C overnight, it was found that, in terms of highest DNA yield, TNES-urea phenol/chloroform extractions performed the best followed by amniocyte phenol/chloroform extractions with kit extractions performing the worst. Be that as it may, it should be noted that all of the extractions did still yield appropriate concentrations of DNA to be used for downstream applications and each has its benefits and limitations. While phenol/chloroform yields higher DNA concentrations, it requires the handling of harmful reagents and is very labour intensive. Kit extractions on the other hand are less time consuming but yield lower quantities of DNA and are also extremely expensive. Since the primary concerns for extracting DNA from the archived scales are low quantity and quality, extractions would likely be done using the TNES-urea phenol/chloroform protocol but at an incubation temperature of 56 °C since this is likely to yield the highest amount of DNA. Because the lengthy process of this method is one of its main drawbacks, future research should determine whether 2 h at this temperature would be sufficient to completely digest the scales as it is when using the extraction kit. It is important to note that any of the protocols discussed in this study can be used to successfully extract DNA from fish scales. The chosen protocol would ultimately be dependent on the specific requirements of the research that is to be completed as well as the accompanying priorities and available reagents.

## Methods

### Sample collection

Samples were collected from a single *A. thorpei* that was sourced from a local fish store where it was stored on ice. This species was chosen due to availability as well as for its scales being similar to those of archived scales that will be used for future research. The *A. thorpei* was scaled completely using a sharp knife and the scales were then stored in a resealable plastic bag (Ziplock) at − 20 °C for DNA extraction. Samples used in the extractions were selected at random from this bag.

### Scale fragmentation using liquid nitrogen

Single scales were placed in a mortar to be frozen with liquid nitrogen in order for them to be ground into a powder for more efficient cell lysis by proteinase K during the incubation stage of the extraction protocol. The liquid nitrogen (Afrox) was removed from the 10 L dewar (Worthington Industries) in which it was stored, using a canister approved for cryogenic liquids, and poured over each scale. After the nitrogen had evaporated, a pestle was used to crush the frozen scales.

### DNA extraction

For each extracted sample, the mean of three measurements that were taken on the Nanodrop 2000 (Thermo Fisher Scientific) was used for statistical analysis (Online Appendix A). One reading from two of the samples was excluded from mean calculations as outliers (Online Appendix A). Of these samples, one was extracted using phenol/chloroform variation 2 (Sample ID T3-S4, excluded reading 2 of 960.6 ng/μl), the other using the DNeasy kit at 56 °C (Sample ID K3-S3, excluded reading 3 of 116.2 ng/μl). All statistical analyses of the measured DNA concentrations were completed using R (Version 3.6.2) and RStudio (Version 1.2.5033) with the packages “readxl” and “car”. Graphs were produced either through Excel or R.

**Figure 6:**
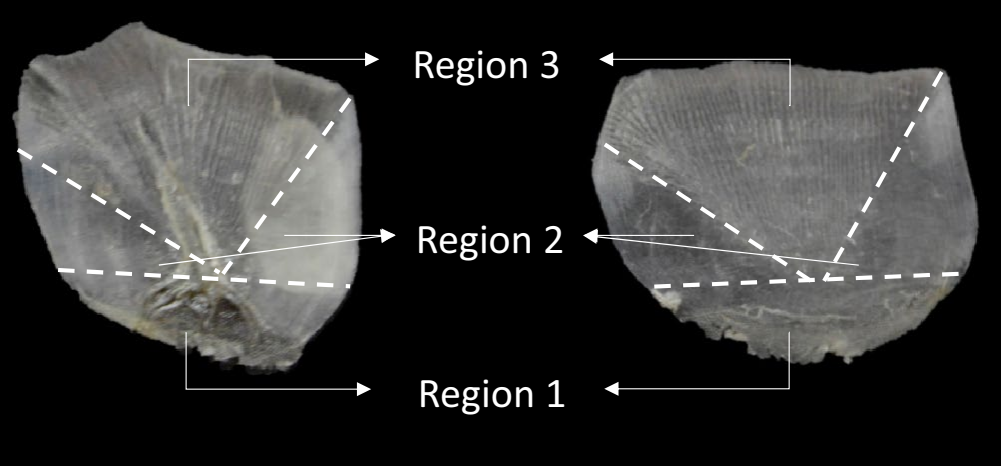
Scales from *Argyrosomus thorpei* divided into specific regions for DNA extraction (Photo by ©L. Shuttleworth, edited in MS PowerPoint Build 14527.20234).

As part of this experiment, whole scales, as well as specific scale regions, were used. How the scales were divided into regions was based on the scale morphology and can be seen in Fig. 6. Whole scales (n = 6) or scale regions (n = 6) were cut into small pieces with sharp, sterilized scissors, and placed in 1.5 ml Eppendorf tubes (J-Plast). Four variations of the standard phenol/chloroform genomic DNA extraction method^40^ were tested based on the methods reported by Wasko et al.^28^ as elaborated on below. Extractions using the DNeasy Blood and Tissue Kit (Qiagen) were also performed to compare DNA extraction efficiency to the phenol/chloroform variations recommended by Wasko et al.^28^.

Three repeats for each extraction protocol were completed on separate days. Following the extraction, a 1.5% agarose gel (Conda Laboratories) stained with GoldView (GeneShun Biotech) was used to assess the presence of DNA. DNA concentration was subsequently quantified using a NanoDrop 2000 Spectrophotometer (Thermo Fisher Scientific).

#### Phenol/chloroform DNA extraction

*Phenol/chloroform DNA extraction variation 1 *Both whole scales and scale regions were placed in 500 μl of a TNES-Urea buffer (10 mM Tris (pH8); 125 mM NaCl; 10 mM EDTA; 0.5% SDS; 4 M urea)^28^ with 30 μl Proteinase K (Inqaba Biotechnical Industries). After mixing using a vortex mixer (Labnet), the scales were left for overnight digestion (> 12 h) at 42 °C. The buffer type as well as incubation temperature used in this variation is the same as that used by Wasko et al.^28^.

Isolation of the DNA was achieved by the addition of two volumes of phenol (Labchem) which had been saturated with 0.1 M Tris (pH8), followed by the addition of a single volume of chloroform:isoamyl alcohol (24:1)(Merck). After the addition of each reagent, the samples were spun in a Spectrafuge 24 D centrifuge (Labnet) at 13,000 rpm for one minute after which the top layer was transferred into a new tube. The DNA was then precipitated with 45 μl of 3 M NaAc (Sigma) and 1000 μl of absolute ethanol (Merck) at − 70 °C for 2 h. Following the allotted time for precipitation to take place, DNA was recovered by 30 min of centrifugation at 13,000 rpm. After removing the supernatant, the DNA pellet was washed with freshly prepared 70% ethanol and centrifuged for another 30 min at 13,000 rpm, the supernatant removed and the pellet allowed to air dry. The DNA was resuspended in 20 μl ddH_2_O. Samples were stored at 4 °C.

*Phenol/chloroform DNA extraction variation 2 *The DNA extraction protocol followed was the same as for Variation 1 except for the digestion temperature which was increased to 56 °C. Only whole scales were used for this part of the experiment.

*Phenol/chloroform DNA extraction variation 3 *For this protocol variation, DNA extractions were completed as in Variation 1 but with the TNES-urea buffer^28^ being substituted with an amniocyte digestion buffer^40^. As with Variation 2, only whole scales were used.

*Phenol/chloroform DNA extraction variation 4 *Finally, a combination Variations 2&3 was followed so that and incubation temperature of 56 °C could be tested while using an amniocyte digestion buffer^40^.

#### Qiagen DNeasy blood and tissue extraction kit

Using the DNeasy Blood and Tissue Kit (Qiagen), extractions from whole scales (n = 6) were performed according to the manufacturer’s instruction at three different incubation temperatures and times. These included (1) 56 °C (2 h), (2) 56 °C (> 12 h) and (3) 42 °C (> 12 h). The DNA was eluted in 100 μl ddH2O and then evaporated to 20 μl for concentration measurements to be comparable with those taken from the phenol/chloroform extractions.

### Confirmation of species ID

Following DNA extraction, PCR of representatives for all the extraction methods used were performed according to procedures followed by Oosthuizen et al.^30^. A 680 bp fragment of the cytochrome oxidase subunit 1 (COI) gene was PCR amplified for species identification. The primers used for species identification were VF2_t1 5’ TGT AAA ACG ACG GCC AGT CAA CCA ACC ACA AAG ACA TTG GCA C 3’ and FishR2_t1 5’ CAG GAA ACA GCT ATG ACA CTT CAG GGT GAC CGA AGA ATC AGA A 3′^41^. Species were positively identified using a standard Nucleotide BLAST search on https://blast.ncbi.nlm.nih.gov/Blast.cgi for highly similar sequences (megablast).

### Ethics approval and consent to participate

This study carried institutional ethics approval under document number NAS314/2020.

## Supplementary Information

Supplementary Information.

## Data Availability

All data generated or analysed during this study are included in this published article [and its supplementary information files].

## Abbreviation

TNES: Tris, NaCl, EDTA, SDS
