# Supplementary material for: Comparing DNA yield from fish scales following different extraction protocols
Source: Sci Rep. 2022 Feb 18;12:2836. doi: 10.1038/s41598-022-06889-y (PMC8857249; doi:10.1038/s41598-022-06889-y)
Supplement: Supplementary file 1 — Supplementary Information. [file 41598_2022_6889_MOESM1_ESM.docx]

**Appendix A:**

DNA concentration of scale samples as measured by using a Nanodrop (Reading 1 – 3) and the mean DNA concentration. Sample ID shows the protocol used (where T = TNES-phenol/chloroform, A = Amniocyte-phenol/chloroform and K = Qiagen DNeasy Blood & Tissue Kits), experimental repeat (T1-T3), scale number (S1-S6) and region (xx-xx.1 – xx-xx.3). Eg. **T1-S1.1** = **TNES**-phenol/chloroform DNA extraction, experimental **repeat 1**, **scale 1** and **region 1**. If xx-xx.1 – xx-xx.3 is absent = whole scale was used as part of th extraction.

|  |  | **DNA concentration** | | | |  | | **DNA concentration** | | | | |  |
| --- | --- | --- | --- | --- | --- | --- | --- | --- | --- | --- | --- | --- | --- |
| **Sample** | **Temp** | **R1** | **R2** | **R3** | **Mean** | **Sample ID** | **Temp** | **R1** | **R2** | **R3** | | **Mean**  **ng/ul** |  |
| **ID** |  | **ng/ul** | **ng/ul** | **ng/ul** | **ng/ul** |  |  | **ng/ul** | **ng/ul** | **ng/ul** | |  |  |
| T1-S1.1 | 42 °C | 8,8 | 7,9 | 8,1 | 8,27 | A1-S6 | 42 °C | 62,6 | 54,2 | | 53,4 | 56,73 |  |
| T1-S1.2 | 42 °C | 8,9 | 6,2 | 6,2 | 7,1 | A2-S1 | 42 °C | 26,7 | 26,9 | | 25,8 | 26,47 |  |
| T1-S1.3 | 42 °C | 9,4 | 8,5 | 10 | 9,3 | A2-S2 | 42 °C | 31,8 | 31 | | 31,6 | 31,47 |  |
| T1-S2.1 | 42 °C | 25,1 | 24,7 | 23 | 24,27 | A2-S3 | 42 °C | 18,2 | 16,8 | | 17,4 | 17,47 |  |
| T1-S2.2 | 42 °C | 9,5 | 10,4 | 9,1 | 9,67 | A2-S4 | 42 °C | 53,7 | 55,3 | | 52,9 | 53,97 |  |
| T1-S2.3 | 42 °C | 20 | 22,9 | 24,1 | 22,33 | A2-S5 | 42 °C | 64,2 | 62,6 | | 60,4 | 62,4 |  |
| T1-S3.1 | 42 °C | 42,1 | 42,5 | 43,4 | 42,67 | A2-S6 | 42 °C | 15,5 | 16,2 | | 18,8 | 16,83 |  |
| T1-S3.2 | 42 °C | 11,9 | 11,5 | 12,8 | 12,07 | A3-S1 | 42 °C | 59,8 | 61,3 | | 54,5 | 58,53 |  |
| T1-S3.3 | 42 °C | 5,5 | 5,7 | 5,8 | 5,67 | A3-S2 | 42 °C | 62,4 | 63,1 | | 63,6 | 63,03 |  |
| T1-S4.1 | 42 °C | 10 | 9,2 | 9 | 9,4 | A3-S3 | 42 °C | 25,1 | 25,4 | | 20,9 | 23,8 |  |
| T1-S4.2 | 42 °C | 7,3 | 7,6 | 7,4 | 7,43 | A3-S4 | 42 °C | 23,1 | 22,8 | | 34 | 26,63 |  |
| T1-S4.3 | 42 °C | 14,7 | 12,6 | 13,6 | 13,63 | A3-S5 | 42 °C | 44,7 | 45,4 | | 43,6 | 44,57 |  |
| T1-S5.1 | 42 °C | 61,5 | 57,9 | 59,1 | 59,5 | A3-S6 | 42 °C | 37,2 | 36,4 | | 37,2 | 36,93 |  |
| T1-S5.2 | 42 °C | 13,8 | 13,3 | 13,5 | 13,53 | A1-S1 | 56 °C | 26,3 | 26,3 | | 24 | 25,53 |  |
| T1-S5.3 | 42 °C | 38 | 38,9 | 39,1 | 38,67 | A1-S2 | 56 °C | 4,7 | 2,2 | | 6,3 | 4,40 |  |
| T1-S6.1 | 42 °C | 29,9 | 28,4 | 28,7 | 29 | A1-S3 | 56 °C | 15,5 | 15,4 | | 15,3 | 15,40 |  |
| T1-S6.2 | 42 °C | 11,6 | 10,6 | 10,8 | 11 | A1-S4 | 56 °C | 7,8 | 7 | | 7,8 | 7,53 |  |
| T1-S6.3 | 42 °C | 9,7 | 9,7 | 10,1 | 9,83 | A1-S5 | 56 °C | 9,4 | 9,7 | | 9,9 | 9,67 |  |
| T2-S1.1 | 42 °C | 35,8 | 37,2 | 38,2 | 37,07 | A1-S6 | 56 °C | 24,5 | 24,2 | | 24,7 | 24,47 |  |
| T2-S1.2 | 42 °C | 14,7 | 14,6 | 14,5 | 14,6 | A2-S1 | 56 °C | 3,5 | 3,4 | | 3,6 | 3,50 |  |
| T2-S1.3 | 42 °C | 29,7 | 31 | 30,6 | 30,43 | A2-S2 | 56 °C | 11 | 11,1 | | 11,4 | 11,17 |  |
| T2-S2.1 | 42 °C | 22,9 | 25,7 | 24,3 | 24,3 | A2-S3 | 56 °C | 5,3 | 5 | | 5,5 | 5,27 |  |
| T2-S2.2 | 42 °C | 21,7 | 21,4 | 22,4 | 21,83 | A2-S4 | 56 °C | 9,4 | 9,1 | | 9,6 | 9,37 |  |
| T2-S2.3 | 42 °C | 34 | 34,8 | 34,1 | 34,3 | A2-S5 | 56 °C | 8,6 | 8,2 | | 8,4 | 8,40 |  |
| T2-S3.1 | 42 °C | 57,1 | 53,1 | 56,7 | 55,63 | A2-S6 | 56 °C | 7,4 | 7,5 | | 7,5 | 7,47 |  |
| T2-S3.2 | 42 °C | 38,6 | 39,9 | 40 | 39,5 | A3-S1 | 56 °C | 7,5 | 7,3 | | 7,3 | 7,37 |  |
| T2-S3.3 | 42 °C | 34,3 | 35,8 | 36,1 | 35,4 | A3-S2 | 56 °C | 8,5 | 8,3 | | 8,4 | 8,40 |  |
| T2-S4.1 | 42 °C | 31,1 | 31,9 | 29,8 | 30,93 | A3-S3 | 56 °C | 5,9 | 5,8 | | 6,3 | 6,00 |  |
| T2-S4.2 | 42 °C | 20,9 | 21,1 | 23,9 | 21,97 | A3-S4 | 56 °C | 36,3 | 36,5 | | 36,4 | 36,40 |  |
| T2-S4.3 | 42 °C | 27,2 | 26,6 | 30 | 27,93 | A3-S5 | 56 °C | 16,5 | 16,6 | | 17,4 | 16,83 |  |
| T2-S5.1 | 42 °C | 21,7 | 19,4 | 18,9 | 20 | A3-S6 | 56 °C | 15,9 | 16,4 | | 15,9 | 16,07 |  |
| T2-S5.2 | 42 °C | 19,6 | 20,1 | 20,5 | 20,07 | K1-S1 | 42 °C | 38,9 | 37,4 | | 35,1 | 37,13 |  |
| T2-S5.3 | 42 °C | 30,5 | 31,6 | 30,8 | 30,97 | K1-S2 | 42 °C | 41,3 | 38,8 | | 38,7 | 39,6 |  |
| T2-S6.1 | 42 °C | 40,1 | 41,6 | 41 | 40,9 | K1-S3 | 42 °C | 18,7 | 18,1 | | 18,8 | 18,53 |  |
| T2-S6.2 | 42 °C | 21,4 | 21,1 | 21 | 21,17 | K1-S4 | 42 °C | 40,5 | 37 | | 36,6 | 38,03 |  |
| T2-S6.3 | 42 °C | 35,2 | 37,3 | 34,9 | 35,8 | K1-S5 | 42 °C | 39,4 | 39 | | 44,5 | 40,97 |  |
| T3-S1.1 | 42 °C | 18,6 | 18,9 | 18,5 | 18,67 | K1-S6 | 42 °C | 36,3 | 34,3 | | 36,4 | 35,67 |  |
| T3-S1.2 | 42 °C | 14 | 13,5 | 13,4 | 13,63 | K2-S1 | 42 °C | 21,5 | 17,5 | | 20,4 | 19,8 |  |
| T3-S1.3 | 42 °C | 16,5 | 16,6 | 16,5 | 16,53 | K2-S2 | 42 °C | 36,7 | 34,2 | | 31,7 | 34,2 |  |
| T3-S2.1 | 42 °C | 61,9 | 61 | 63,4 | 62,1 | K2-S3 | 42 °C | 30 | 28,1 | | 28,1 | 28,73 |  |
| T3-S2.2 | 42 °C | 18,5 | 18,7 | 18,9 | 18,7 | K2-S4 | 42 °C | 32,9 | 28,9 | | 28,6 | 30,13 |  |
| T3-S2.3 | 42 °C | 26,6 | 26,8 | 25,7 | 26,37 | K2-S5 | 42 °C | 30,5 | 29,7 | | 27,8 | 29,33 |  |
| T3-S3.1 | 42 °C | 25,1 | 24,4 | 24,6 | 24,7 | K2-S6 | 42 °C | 32,7 | 30,1 | | 29,6 | 30,8 |  |
| T3-S3.2 | 42 °C | 38,4 | 38,5 | 37,8 | 38,23 | K3-S1 | 42 °C | 28,4 | 26,8 | | 25,4 | 26,87 |  |
| T3-S3.3 | 42 °C | 20,7 | 20,9 | 21,1 | 20,9 | K3-S2 | 42 °C | 24,1 | 23,6 | | 23,1 | 23,6 |  |
| T3-S4.1 | 42 °C | 12,9 | 12,1 | 11,9 | 12,3 | K3-S3 | 42 °C | 27,3 | 28,5 | | 29 | 28,27 |  |
| T3-S4.2 | 42 °C | 20,5 | 21,3 | 19,9 | 20,57 | K3-S4 | 42 °C | 24,7 | 23,5 | | 22,4 | 23,53 |  |
| T3-S4.3 | 42 °C | 26,6 | 26,1 | 26,1 | 26,27 | K3-S5 | 42 °C | 26 | 24,2 | | 22 | 24,07 |  |
| T3-S5.1 | 42 °C | 39,6 | 38 | 37 | 38,2 | K3-S6 | 42 °C | 24,5 | 26,7 | | 22,9 | 24,7 |  |
| T3-S5.2 | 42 °C | 15,6 | 15,2 | 15,4 | 15,4 | K1-S1 | 56 °C (2 hrs) | 53,2 | 53,5 | | 55,6 | 54,1 |  |
| T3-S5.3 | 42 °C | 12,9 | 12,7 | 12,6 | 12,73 | K1-S2 | 56 °C (2 hrs) | 52,6 | 49,4 | | 48,4 | 50,13 |  |
| T3-S6.1 | 42 °C | 43,1 | 41,4 | 42 | 42,17 | K1-S3 | 56 °C (2 hrs) | 58,6 | 56,9 | | 54 | 56,5 |  |
| T3-S6.2 | 42 °C | 14 | 14 | 13,6 | 13,87 | K1-S4 | 56 °C (2 hrs) | 54,1 | 53,9 | | 55,2 | 54,4 |  |
| T3-S6.3 | 42 °C | 40,1 | 37 | 40 | 39,03 | K1-S5 | 56 °C (2 hrs) | 44,3 | 42,3 | | 43,5 | 43,37 |  |
| T1-S1 | 42 °C | 98,5 | 98,4 | 96,6 | 97,83 | K1-S6 | 56 °C (2 hrs) | 53 | 50,7 | | 51 | 51,57 |  |
| T1-S2 | 42 °C | 102,3 | 93,2 | 94,1 | 96,53 | K2-S1 | 56 °C (2 hrs) | 27,5 | 26,2 | | 26,8 | 26,83 |  |
| T1-S3 | 42 °C | 57,5 | 58,5 | 61,5 | 59,17 | K2-S2 | 56 °C (2 hrs) | 39,8 | 41,3 | | 41,5 | 40,87 |  |
| T1-S4 | 42 °C | 81,6 | 83,6 | 81,3 | 82,17 | K2-S3 | 56 °C (2 hrs) | 42,5 | 38,7 | | 40,6 | 40,6 |  |
| T1-S5 | 42 °C | 72 | 73,4 | 72,7 | 72,7 | K2-S4 | 56 °C (2 hrs) | 45,3 | 42,5 | | 43,4 | 43,73 |  |
| T1-S6 | 42 °C | 21,4 | 21 | 24,4 | 22,27 | K2-S5 | 56 °C (2 hrs) | 37,5 | 37,5 | | 33,7 | 36,23 |  |
| T2-S1 | 42 °C | 66,8 | 78,6 | 61,9 | 69,1 | K2-S6 | 56 °C (2 hrs) | 35,2 | 32,5 | | 33,1 | 33,6 |  |
| T2-S2 | 42 °C | 66,4 | 70,9 | 66,8 | 68,03 | K3-S1 | 56 °C (2 hrs) | 30,1 | 31,9 | | 31,8 | 31,27 |  |
| T2-S3 | 42 °C | 98,9 | 96,6 | 101 | 98,83 | K3-S2 | 56 °C (2 hrs) | 24,3 | 23,9 | | 22 | 23,4 |  |
| T2-S4 | 42 °C | 50,5 | 56,6 | 57,9 | 55 | K3-S3 | 56 °C (2 hrs) | 31,3 | 31,7 | | 32,1 | 31,7 |  |
| T2-S5 | 42 °C | 15,2 | 13,5 | 13,9 | 14,2 | K3-S4 | 56 °C (2 hrs) | 42,1 | 42,2 | | 40,9 | 41,73 |  |
| T2-S6 | 42 °C | 47,9 | 48,4 | 51,7 | 49,33 | K3-S5 | 56 °C (2 hrs) | 35,2 | 36,8 | | 37,2 | 36,4 |  |
| T3-S1 | 42 °C | 130,2 | 135,3 | 137,6 | 134,37 | K3-S6 | 56 °C (2 hrs) | 22,9 | 22,5 | | 21,1 | 22,17 |  |
| T3-S2 | 42 °C | 102,1 | 100,6 | 106,6 | 103,1 | K1-S1 | 56 °C | 19,8 | 17 | | 19,4 | 18,73 |  |
| T3-S3 | 42 °C | 80,1 | 76,1 | 79,2 | 78,47 | K1-S2 | 56 °C | 29,5 | 35,3 | | 34,7 | 33,17 |  |
| T3-S4 | 42 °C | 102,4 | 104,8 | 104,8 | 104 | K1-S3 | 56 °C | 19,3 | 19,4 | | 18,7 | 19,13 |  |
| T3-S5 | 42 °C | 196,3 | 185,3 | 177,9 | 186,5 | K1-S4 | 56 °C | 39,8 | 35,9 | | 37,6 | 37,77 |  |
| T3-S6 | 42 °C | 75,5 | 80,7 | 82,6 | 79,6 | K1-S5 | 56 °C | 23,5 | 22,8 | | 22,9 | 23,07 |  |
| T1-S1 | 56 °C | 150,8 | 153,6 | 156,4 | 170,43 | K1-S6 | 56 °C | 29 | 30,8 | | 27,2 | 29 |  |
| T1-S2 | 56 °C | 55,2 | 53,1 | 53,6 | 53,97 | K2-S1 | 56 °C | 43,4 | 41,8 | | 40 | 41,73 |  |
| T1-S3 | 56 °C | 178,1 | 180,6 | 181 | 175,8 | K2-S2 | 56 °C | 29 | 27,3 | | 28,7 | 28,33 |  |
| T1-S4 | 56 °C | 78,7 | 83,3 | 79,7 | 80,57 | K2-S3 | 56 °C | 31,7 | 29,8 | | 34,2 | 31,9 |  |
| T1-S5 | 56 °C | 117,4 | 118,9 | 117,8 | 170,63 | K2-S4 | 56 °C | 44,9 | 44,2 | | 41,2 | 43,43 |  |
| T1-S6 | 56 °C | 100,6 | 103,7 | 101,8 | 100,5 | K2-S5 | 56 °C | 44,3 | 42,8 | | 40 | 42,37 |  |
| T2-S1 | 56 °C | 127,8 | 130,3 | 129 | 129,03 | K2-S6 | 56 °C | 32,5 | 31,6 | | 31,7 | 31,93 |  |
| T2-S2 | 56 °C | 75,6 | 75,4 | 77,5 | 76,17 | K3-S1 | 56 °C | 31,5 | 28,1 | | 41,8 | 33,8 |  |
| T2-S3 | 56 °C | 96,7 | 99,3 | 100 | 98,67 | K3-S2 | 56 °C | 54,4 | 49,2 | | 52,3 | 51,97 |  |
| T2-S4 | 56 °C | 100 | 95,5 | 99,9 | 98,47 | K3-S3 | 56 °C | 37,2 | 32,4 | | 116,2 | 61,93 |  |
| T2-S5 | 56 °C | 131,7 | 135,6 | 133,2 | 133,5 | K3-S4 | 56 °C | 36,5 | 35,5 | | 36,6 | 36,2 |  |
| T2-S6 | 56 °C | 152,4 | 154,7 | 156 | 154,37 | K3-S5 | 56 °C | 25,1 | 22,5 | | 20,2 | 22,6 |  |
| T3-S1 | 56 °C | 85,5 | 85,2 | 85,4 | 85,37 | K3-S6 | 56 °C | 31,5 | 30,5 | | 27,4 | 29,8 |  |
| T3-S2 | 56 °C | 88,4 | 150,2 | 110,5 | 116,37 | K2-S4 | 56 °C | 44,9 | 44,2 | | 41,2 | 43,43 |  |
| T3-S3 | 56 °C | 74,7 | 72,3 | 73 | 73,33 | K2-S5 | 56 °C | 44,3 | 42,8 | | 40 | 42,37 |  |
| T3-S4 | 56 °C | 387,5 | 960,6 | 293,7 | 547,27 | K2-S6 | 56 °C | 32,5 | 31,6 | | 31,7 | 31,93 |  |
| T3-S5 | 56 °C | 173,4 | 169,6 | 184,9 | 175,97 | K3-S1 | 56 °C | 31,5 | 28,1 | | 41,8 | 33,8 |  |
| T3-S6 | 56 °C | 137,4 | 137,4 | 128,4 | 134,4 | K3-S2 | 56 °C | 54,4 | 49,2 | | 52,3 | 51,97 |  |
| A1-S1 | 42 °C | 9,8 | 9,3 | 11,4 | 10,17 | K3-S3 | 56 °C | 37,2 | 32,4 | | 116,2 | 61,93 |  |
| A1-S2 | 42 °C | 12 | 14,6 | 9,6 | 12,07 | K3-S4 | 56 °C | 36,5 | 35,5 | | 36,6 | 36,2 |  |
| A1-S3 | 42 °C | 26,5 | 28,6 | 26,1 | 27,07 | K3-S5 | 56 °C | 25,1 | 22,5 | | 20,2 | 22,6 |  |
| A1-S4 | 42 °C | 15,2 | 13,6 | 12,4 | 13,73 | K3-S6 | 56 °C | 31,5 | 30,5 | | 27,4 | 29,8 |  |
| A1-S5 | 42 °C | 25,5 | 50,1 | 22,5 | 32,7 |  |  |  |  | |  |  |  |

**Appendix B:**

R code used for statistical analysis and creation of Figures 3, 5 & 6.

####Packages####

library(car)

library(readxl)

####TNES Temp Comparison####

TempTNES

TempTNES$Temp <- as.factor(TempTNES$Temp)

is.factor(TempTNES$Temp)

is.numeric(TempTNES$Concentration)

shapiro.test(TempTNES$Concentration)

wilcox.test(TempTNES$Concentration~TempTNES$Temp)

names1<-c("42 °C", "56 °C")

Boxplot1 <- boxplot(Concentration~Temp, data=TempTNES, ylab="Concentration (ng/μl)", xlab="Temperature", names=names1)

Boxplot1

####Comparing Regions####

Regions

Regions$Region <- as.factor(Regions$Region)

is.factor(Regions$Region)

is.numeric(Regions$Concentration)

Model1 <- lm(Concentration~Region, data= Regions)

Model1

summary(Model1)

ANOVA1 <-aov(Model1)

TUKEY1 <- TukeyHSD(ANOVA1, conf.level = 0.99)

TUKEY1

plot(TUKEY1)

####Comparing TNES to Amnio at 42 degrees####

AmnioTNES

AmnioTNES$Buffer <- as.factor(AmnioTNES$Buffer)

is.factor(AmnioTNES$Buffer)

is.numeric(AmnioTNES$Concentration)

shapiro.test(AmnioTNES$Concentration)

wilcox.test(AmnioTNES$Concentration~AmnioTNES$Buffer)

names2<-c("TNES", "Amniocyte")

AmnioTNES$Buffer <- factor(AmnioTNES$Buffer , levels=c("TNES", "AMNIO"))

Boxplot2 <- boxplot(Concentration~Buffer, data=AmnioTNES, ylab="Concentration (ng/μl)", xlab="Buffer Type", names=names2)

Boxplot2

####Comparing the incubation time using the kit at 56 degrees####

TimeKit

TimeKit$Hours <- as.factor(TimeKit$Hours)

is.factor(TimeKit$Hours)

is.numeric(TimeKit$Concentration)

shapiro.test(TimeKit$Concentration)

t.test(TimeKit$Concentration~TimeKit$Hours)

names3<-c("2", "12")

Boxplot3 <- boxplot(Concentration~Hours, data=TimeKit, ylab="Concentration (ng/μl)", xlab="Incubation Time (Hours)", names=names3)

Boxplot3

####Comparing Temperatures using the kit####

TemperatureKit

TemperatureKit$Temp <- as.factor(TemperatureKit$Temp)

is.factor(TemperatureKit$Temp)

is.numeric(TemperatureKit$Concentration)

shapiro.test(TemperatureKit$Concentration)

t.test(TemperatureKit$Concentration~TemperatureKit$Temp)

names4<-c("56 °C", "42 °C")

TemperatureKit$Temp <- factor(TemperatureKit$Temp , levels=c("Degrees56", "Degrees42"))

Boxplot4 <- boxplot(Concentration~Temp, data=TemperatureKit, ylab="Concentration (ng/μl)", xlab="Temperature", names=names4)

Boxplot4

####Comparing all 3 protocols at 42 degrees####

Protocol

Protocol$Protocol <- as.factor(Protocol$Protocol)

is.factor(Protocol$Protocol)

is.numeric(Protocol$Concentration)

Model2 <- lm(Concentration~Protocol, data= Protocol)

Model2

ANOVA2 <-aov(Model2)

TUKEY2 <- TukeyHSD(ANOVA2, conf.level = 0.99)

TUKEY2

plot(TUKEY2)

names5<-c("TNES-Phenol/Chloroform" , "Amniocyte-Phenol/Chloroform", "Kit")

Protocol$Protocol <- factor(Protocol$Protocol , levels=c("TNES", "AMNIO" , "KIT"))

Boxplot5 <- boxplot(Concentration~Protocol, data=Protocol, ylab="Concentration (ng/μl)", xlab="Protocol", names=names5)

Boxplot5

####Compariong temps with Amnio buffer####

is.factor(Amnio$Temp)

Amnio$Temp <- as.factor(Amnio$Temp)

is.numeric(Amnio$Concentration)

shapiro.test(Amnio$Concentration)

wilcox.test(Amnio$Concentration~Amnio$Temp)

names1<-c("42 °C", "56 °C")

Boxplot6 <- boxplot(Concentration~Temp, data=Amnio, ylab="Concentration (ng/μl)",

xlab="Temperature", names=names1, col="white")
